# Novel pneumococcal capsule type 33E results from the inactivation of glycosyltransferase WciE in vaccine type 33F

**DOI:** 10.1016/j.jbc.2023.105085

**Published:** 2023-07-24

**Authors:** Feroze A. Ganaie, Jamil S. Saad, Stephanie W. Lo, Lesley McGee, Andries J. van Tonder, Paulina A. Hawkins, Juan J. Calix, Stephen D. Bentley, Moon H. Nahm

**Affiliations:** 1 Division of Pulmonary/Allergy/Critical Care, Department of Medicine, University of Alabama at Birmingham, Birmingham, Alabama, USA; 2 Department of Microbiology, University of Alabama at Birmingham, Birmingham, Alabama, USA; 3 Parasites and Microbes, Wellcome Sanger Institute, Cambridge, United Kingdom; 4 Respiratory Diseases Branch, Centers for Disease Control and Prevention, Atlanta, Georgia, USA; 5 Department of Veterinary Medicine, University of Cambridge, Cambridge, United Kingdom; 6 CDC Foundation, Atlanta, Georgia, USA; 7 Division of Infectious Diseases, Department of Medicine, University of Alabama at Birmingham, Birmingham, Alabama, USA

**Keywords:** *Streptococcus pneumoniae*, capsule polysaccharide, serotype, vaccine, capsule type, vaccine escape

## Abstract

The polysaccharide (PS) capsule is essential for immune evasion and virulence of *Streptococcus pneumoniae*. Existing pneumococcal vaccines are designed to elicit anticapsule antibodies; however, the effectiveness of these vaccines is being challenged by the emergence of new capsule types or variants. Herein, we characterize a newly discovered capsule type, 33E, that appears to have repeatedly emerged from vaccine type 33F *via* an inactivation mutation in the capsule glycosyltransferase gene, *wciE*. Structural analysis demonstrated that 33E and 33F share an identical repeat unit backbone [→5)-β-D-Gal*f*2Ac-(1→3)-β-D-Gal*p*-(1→3)-α-D-Gal*p*-(1→3)-β-D-Gal*f*-(1→3)-β-D-Glc*p*-(1→], except that a galactose (α-D-Gal*p*) branch is present in 33F but not in 33E. Though the two capsule types were indistinguishable using conventional typing methods, the monoclonal antibody Hyp33FM1 selectively bound 33F but not 33E pneumococci. Further, we confirmed that *wciE* encodes a glycosyltransferase that catalyzes the addition of the branching α-D-Gal*p* and that its inactivation in 33F strains results in the expression of the 33E capsule type. Though 33F and 33E share a structural and antigenic similarity, our pilot study suggested that immunization with a 23-valent pneumococcal PS vaccine containing 33F PS did not significantly elicit cross-opsonic antibodies to 33E. New conjugate vaccines that target capsule type 33F may not necessarily protect against 33E. Therefore, studies of new conjugate vaccines require knowledge of the newly identified capsule type 33E and reliable pneumococcal typing methods capable of distinguishing it from 33F.

*Streptococcuspneumoniae* (pneumococcus) is a Gram-positive pathobiont that normally colonizes the human nasopharynx but can also cause pneumonia and severe disease upon invasion of sterile sites, such as the middle ear, bloodstream, and meninges (1, 2). The World Health Organization estimates that pneumococci are responsible for more than 300,000 annual deaths among children aged under 5 years (3). Its survival in human hosts is facilitated by the production of a polysaccharide (PS) capsule that shields the bacteria during nasopharyngeal colonization and prevents opsonophagocytosis (4). Antibodies targeting the immunodominant capsule can prevent colonization and disease, and this acquired immunity has likely contributed to the evolution of over 100 antigenically diverse capsule types among pneumococci (5, 6, 7, 8, 9, 10). Ultimately defined by the unique biochemical structure of their PS, capsule types are conventionally assigned a “serotype” according to reactivity with reference antisera, and serotypes sharing antigenic properties are organized into “serogroups.”

Most pneumococci produce capsule through a highly organized, Wzy-dependent process mediated by type-specific genes located in the capsule synthesis (*cps*) locus (11). Briefly, a type-specific oligosaccharide repeat unit (RU) is sequentially synthesized on a lipid carrier *via* the coordinated activity of *cps* glycosyltransferases (GTs). Some GTs utilize nonhousekeeping donor substrates that must be synthesized by other *cps* enzymes, *e.g*., the pneumococcal GT WciB uses UDP-galactofuranose (Gal*f*) made by the *cps*-encoded Glf synthetase, etc. Completed RUs are exported to the bacterial surface by a Wzx flippase and polymerized into glycan chains by a Wzy polymerase. The glycans can also be modified by, often multiple, *cps* O-acetyltransferases. Ultimately, mature glycan chains are covalently anchored to the cell wall, forming a glycocalyx that envelops the entire bacterium. Because *cps* genes mediate PS structure, *cps* locus nucleotide identity can also be used to predict a strain’s capsule type (12), a process herein called “*cps* typing”.

Pneumococcal serotyping and *cps* typing primarily rely on predefined antigenic markers or reference *cps* locus sequences, respectively—many of which were described decades ago from an arbitrary subset of strains. As a result, biochemically distinct capsule variants with similar antigenic profile or *cps* locus sequences can be mistakenly grouped as a single capsule type, masking relevant distinctions in their epidemiological behaviors (5, 8, 13). Accurate capsule typing methods have become especially valuable in evaluating the impact of pneumococcal immunization efforts. Widespread use of pneumococcal conjugate vaccines (PCVs) containing PS of the most clinically relevant capsule types has drastically reduced pneumococcal disease (14, 15, 16, 17) but has resulted in a relative upsurge in the prevalence by nonvaccine capsule types (6, 18, 19). For example, capsule type (serotype) 33F, which was not included in earlier PCV formulations (*i.e.*, PCV7, PCV10, and PCV13), has become one of the most predominant serotypes causing pneumococcal disease globally (20, 21). Consequently, type 33F PS is now included in PCV15 and PCV20 licensed in 2021 (22).

33F capsule and the closely related 33A capsule are identical, except for the presence of 5,6-O-acetylation of the reducing-end Gal*f* in 33A, but not in 33F (23) (Fig. 1*A*). This capsule PS modification in 33A, herein called “factor 20b” due to its association with factor serum 20b reactivity (23), is putatively mediated by the intact *cps*-encoded O-acetyltransferase WcjE. However, 33F *cps* loci harbor a *wcjE* pseudogene (24, 25) and, thus, distinctively lack factor 20b. Recent independent *cps* typing studies identified a novel 33F-like genetic variant, herein referred to as the “33F-1” (24, 26). The 33F-1 *cps* loci have been reported in multiple isolates from Fiji and Mongolia (26) and more recently from isolates recovered from 10 separate countries (24) (Table S1). Compared to reference 33F sequences, 33F-1 *cps* loci harbor a putative O-acetyltransferase *wcyO* instead of *wcjE* pseudogenes (24, 26). Though 33F-1 strains were repeatedly typed as “33F” according to conventional serotyping methods (24, 26), their capsule PS structures have not been evaluated. Here, we performed in-depth genetic and phenotypic characterization of 33F-1 isolates, resulting in the identification of three genetic subtypes, herein named “33F-1a, 33F-1b, and 33F-1c”. 33F-1a produces capsule PS identical to 33F, while 33F-1b exhibits a similar *cps* gene content to 33F, suggesting the production of a similar capsule PS. In contrast, 33F-1c has a nonsense mutation in the GT gene *wciE*, resulting in the loss of a branching galactopyranose (Gal*p*) on their capsule PS. 33F-1c is antigenically distinguishable from 33F using a serotyping monoclonal antibody (mAb) and, compared to 33F, is less effectively targeted by human sera following immunization with 33F PS. Thus the third genetic subtype, 33F-1c, expresses an antigenically distinct novel capsule type which is named 33E.

**Figure 1:**
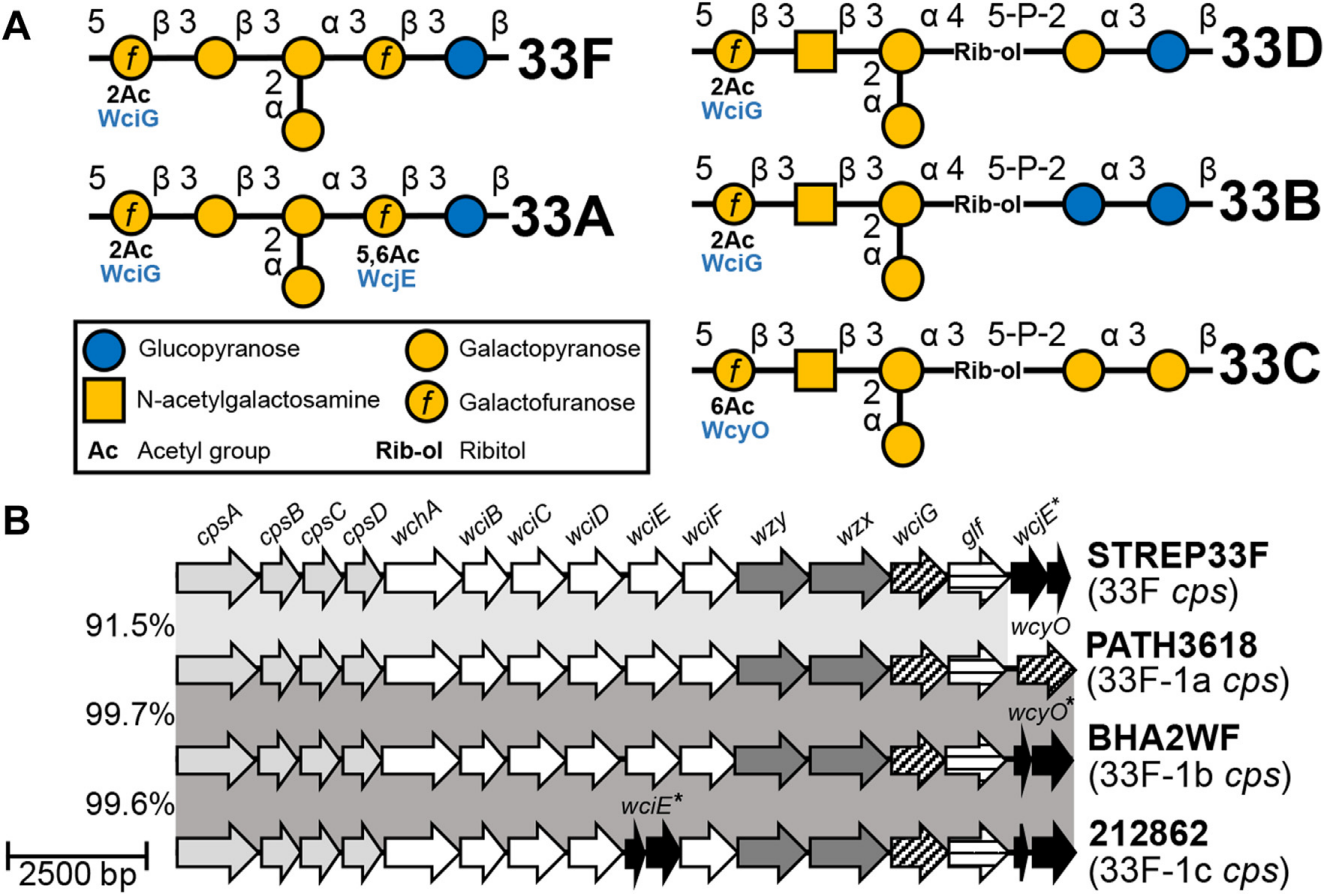
**Capsule polysaccharide structures and *cps* loci of serogroup 33 capsule types.***A*, Symbol Nomenclature for Glycans diagrams of elucidated serogroup 33 PS repeat unit structures. *cps*-encoded O-acetyltransferases are listed in *blue text* underneath their assigned O-acetyl substitutions. *B*, alignment of representative 33F and 33F-1 variant *cps* loci (*i.e.*, 33F-1a, 33F-1b, and 33F-1c), as noted in parentheses below the strain name to the right. Genes encoding glycosyltransferases (*white arrows*), Wzx/Wzy enzymes (*dark gray arrows*), carbohydrate synthetases (*horizontally striped arrows*), highly conserved biosynthetic elements (*light gray arrows*), O-acetyltransferases (*vertical striped arrows*), and pseudogene elements (*black arrows*) are labeled at the *top*. *Asterisks* denote alleles encoding pseudogenes. The percent identity of regions highlighted by *gray boxes* is noted to the *left*, between each row. *cps*, capsule synthesis locus; PS, polysaccharide.

## Results

### 33F-1 cps locus variants contain variably inactivated wcyO and wciE genes

We reanalyzed 33F-1 *cps* loci by alignment to a reference 33F *cps* locus from strain STREP33F. STREP33F is a laboratory-derived serotype 33F strain routinely used to evaluate serological response to immunization (27). Whole genome sequencing of STREP33F revealed it contains a *cps* locus that shares 99.9% identity with the reference 33F *cps* locus from strain 3084/37 (GenBank accession no. CR931702) (25), except for a different *wcjE* pseudogene allele. As reported previously, while 33F-1 *cps* loci shared >99.5% nucleotide identity among themselves, they share only 91 to 92% identity with canonical 33F *cps* loci (*i.e*., STREP33F) upstream of the *wcjE* pseudogene (Fig. 1*B*). As noted above, all 33F-1 strains harbored a *wcyO* allele instead of the *wcjE* pseudogene present in the STREP33F *cps* locus.

Further comparative analysis of 33F-1 *cps* loci identified three genetic subtypes, herein referred to as 33F-1a, -1b, and -1c according to the content of intact *cps* genes (Fig. 1*B*). 33F-1a *cps* loci, represented by the genome of GPS_US_PATH3618 (herein called “PATH3618”, NCBI accession no. ERR750820), contain a putatively intact *wcyO* allele that shares 99.3% identity with the *wcyO* allele from the reference 33C *cps* locus (25). Since WcyO putatively mediates 6-O-acetylation of the nonreducing end Gal*f* in multiple capsule types, including serotype 33C (28) (Fig. 1*A*), capsule PS encoded by 33F-1a loci may have differing O-acetylation patterns to canonical 33F PS. Most 33F-1 isolates contained a 33F-1b *cps* locus, represented by the genome of GPS_BHA2WF (NCBI accession no. MK606435), which contains a *wcyO* pseudogene. The 33F-1b *cps* loci harbor either a single nucleotide “T” insertion (in isolates Fiji) or a single nucleotide “A” deletion (in all other isolates) frame-shift mutations in their *wcyO* pseudogenes. Lastly, two isolates contained a 33F-1c *cps* locus, represented by the genome of strain 2009212862 (herein called “212862”, GenBank accession no. ERR433945). In addition to the *wcyO* pseudogene, the 33F-1c locus contains a nonsense mutation in the putative GT gene *wciE*, which results in a truncated pseudogene encoding only the first 91 of 323 amino acids in 212862. The *cps* locus of other 33F-1c strain, GPS_NZ_SPN11350 (GenBank accession no. ERR1788088) contained a nonsense mutation in *wciE* at a different site that results in a truncated pseudogene encoding only the first 130 amino acids; however, its *wcyO* gene is intact. Given these differences in *cps* gene content, we hypothesized the presence of three unique capsule PS structures among 33F-1 isolates.

### Biochemical structure of capsule PS purified from 33F-1c strain differs from canonical 33F capsule PS

**Figure 2:**
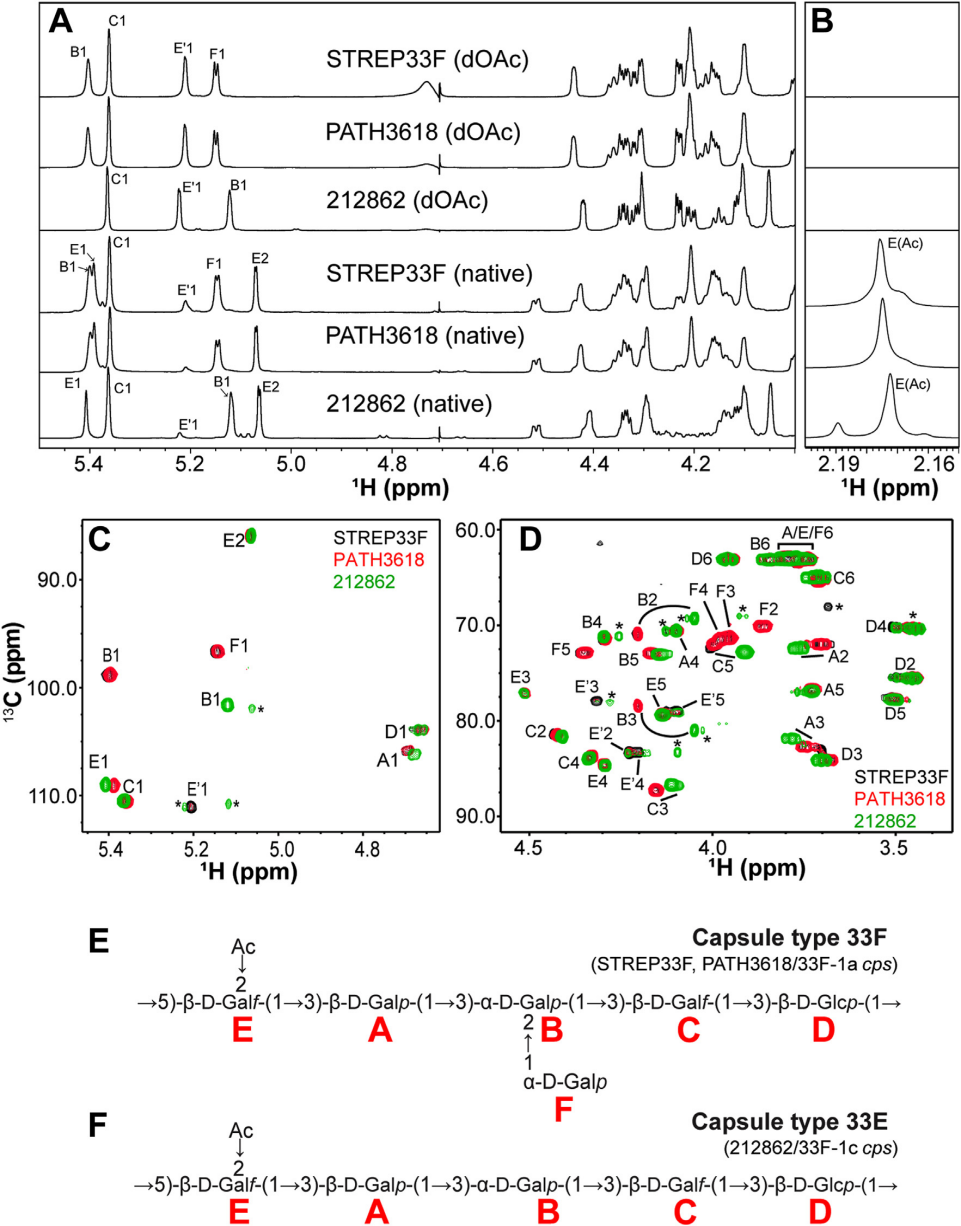
**Biochemical analysis of capsule polysaccharide produced by 33F *cps* type variants.***A* and *B*, ^1^H NMR spectra showing the anomeric (*panel A*) and acetyl (*panel B*) regions of de-O-acetylated (dOAc) and native PS purified from STREP33F (33F), PATH3618 (33F-1a), and 212862 (33F-1c). The peaks for A1 and D1 are obscured by the HOD peak and therefore not assignable in the ^1^H NMR spectrum. *C* and *D*, overlay of ^1^H-^13^C HMQC NMR spectra showing the anomeric (*panel C*) and ring (*panel D*) regions for STREP33F (*black*), PATH3618 (*red*), and 212862 (*green*) PS. Labels in *Panels A*–*D* denote signals assigned to the corresponding residues “*A*–*F*”, as labeled in *panels E* and *F*, as well as in Table 1. Residue Eꞌ represents the de-O-acetylated form of glycosyl residue E. *E* and *F*, structures of 33F (*panelE*) and 33E (*panelF*) PS. *Red letters* denote the residue name assigned to each monosaccharide in NMR analyses. Strains/genotypes producing each PS are listed in parentheses. *cps*, capsule synthesis locus; NMR, nuclear magnetic resonance; PS, polysaccharide.

To examine this hypothesis, we analyzed de-O-acetylated (dOAc, *via* mild alkali hydrolysis) and native PS purified from pneumococcal strains STREP33F (33F), PATH3618 (33F-1a) and 212862 (33F-1c) by nuclear magnetic resonance (NMR). Our structural analysis did not include 33F-1b isolates owing to their unavailability and the fact that their *cps* loci exhibited similar gene content to 33F and thus should produce the same capsule PS structure. ^1^H NMR spectra of deOAc PS purified from STREP33F and PATH3618 were indistinguishable (Fig. 2*A*). While the spectra of these PS contained four major peaks in the anomeric region between 5.0 and 5.5 ppm, the ^1^H NMR spectra of dOAc PS from 212862 only contained three peaks. The spectra of native PS of STREP33F and PATH3618 were also indistinguishable. Though 212862 native PS still lacked a major anomeric peak, we observed similar changes in the spectra of all three native PS samples compared to their dOAc counterparts, *e.g.*, the acquisition of a major peak at 5.07 ppm (Fig. 2*A*) and a single major resonance in the O-acetyl methyl region at 2.17 to 2.18 ppm (Fig. 2*B*). Altogether, this suggested all three PS samples contained an O-acetate substitution at a single shared site, despite the 212862 PS RU presumptively lacking a monosaccharide residue.

Indeed, we assigned all ^1^H and ^13^C NMR signals in the heteronuclear multiple quantum coherence spectra (Fig. 2, *C* and *D*) to either six (in STREP33F or PATH3618) or five (in 212862) spin systems labeled as residues A-F (Fig. 2, *E* and *F*). Complete assignment of ^1^H and ^13^C chemical shifts by homonuclear and heteronuclear 2D NMR experiments (Table 1) revealed that all three PS capsules were composed of a backbone containing one Glc*p* (residue D), two Gal*f* (residues C and E), and two Gal*p* (residues A and B). However, the STREP33F and PATH3618 PS RU contained a branching α-D-Gal*p* (residue F), which was missing from the 212862 PS RU. Native PS of all three samples contained a single O-acetylation on the 2 position of residue E. The presence of 2-O-acetylated Gal*f* (residue E) was consistent with the ^1^H spectral changes observed between deOAc and native PS samples (Fig. 2*A*). Notably, we did not detect any NMR evidence of 6-O-acetylation, as observed in other *wcyO*- or *wcjE*-associated serogroup 33 capsule types (23, 28, 29). In summary, STREP33F and PATH3618 produce capsule PS with structures identical to canonical type 33F (Fig. 2*E*), while 212862 produces a novel capsule PS structure (Fig. 2*F*), herein called 33E. We conclude that 33F-1a and 33F-1b genetic subtypes that contain an intact *wciE* gene produce 33F capsule PS, while the 33F-1c genetic subtype, which contains a *wciE* pseudogene, produces 33E capsule PS.

**Table 1:** ^1^H and^13^C chemical shifts (ppm) of serotypes 33F and 33E obtained at 35 °C

Residue	Label	H1/C1	H2/C2	H3/C3	H4/C4	H5/C5	H6a,H6b/C6	O-Ac
Serotype 33F								
→3-β-D-Gal*p*-(1→	A	4.69/105.8	3.70/72.1	3.70/83.1	4.10/70.6	3.73/76.8	3.76,3.78/63.0	
→2,3-α-D-Gal*p*-(1→	B	5.40/98.8	4.20/70.86	4.20/78.3	4.29/71.4	4.17/73.0	3.80,3.85/63.0	
→3-β-D-Gal*f*-(1→	C	5.36/110.5	4.42/81.5	4.15/87.2	4.33/83.9	4.00/72.1	3.70,3.71/65.3	
→3-β-D-Glc*p*-(1→	D	4.67/103.9	3.49/75.5	3.68/84.3	3.50/70.2	3.51/77.8	3.94,3.95/63.0	
→5-β-D-Gal*f*-(1→	E	5.39/109.0	5.07/86.0	4.51/77.0	4.30/84.7	4.13/79.3	3.74,3.75/63.1	2.17/22.5
	Eꞌ	5.21/111.1	4.22/83.4	4.30/77.8	4.19/83.5	4.10/79.1	3.74,3.75/63.1	
→α-D-Gal*p*-(1→	F	5.14/96.8	3.86/70.0	3.95/71.3	3.98/71.7	4.35/72.9	3.78,3.79/63.0	
Serotype 33E								
→3-β-D-Gal*p*-(1→	A	4.68/106.1	3.76/72.5	3.78/81.9	4.10/70.6	3.73/76.8	3.76,3.78/63.0	
→3-α-D-Gal*p*-(1→	B	5.11/101.6	4.05/69.3	4.05/81.0	4.29/71.4	4.14/73.2	3.80,3.85/63.0	
→3-β-D-Gal*f*-(1→	C	5.36/110.4	4.40/81.7	4.11/86.7	4.34/84.1	3.91/72.9	3.70,3.73/65.0	
→3-β-D-Glc*p*-(1→	D	4.67/103.9	3.49/75.6	3.70/84.2	3.49/70.4	3.50/78.0	3.94,3.95/63.0	
→5-β-D-Gal*f*-(1→	E	5.40/109.0	5.06/86.0	4.51/77.0	4.30/84.7	4.13/79.3	3.74,3.75/63.1	2.17/22.5
	Eꞌ	na	4.22/83.4	4.30/77.8	4.19/83.5	4.10/79.1	3.74,3.75/63.1	

Each carbohydrate residue is labeled with a unique letter for 33F (A-F) and 33E (A–E). Residue Eꞌ represents the de-O-acetylated form of glycosyl residue E. Proton and carbon atoms are indicated by letters H and C, respectively, and the numbers associated with them indicate their respective position. A slash separates the proton and carbon chemical shifts (ppm). For each residue, the table shows the chemical shifts of every proton and carbon molecule attached to it at different positions.

Abbreviations: na, not assigned due to overlap with impurity and/or weak intensity; O-Ac, O-acetylation.

### Inactivation of wciE results in the loss of a branching α-D-Galp on the capsule PS RU

**Figure 3:**
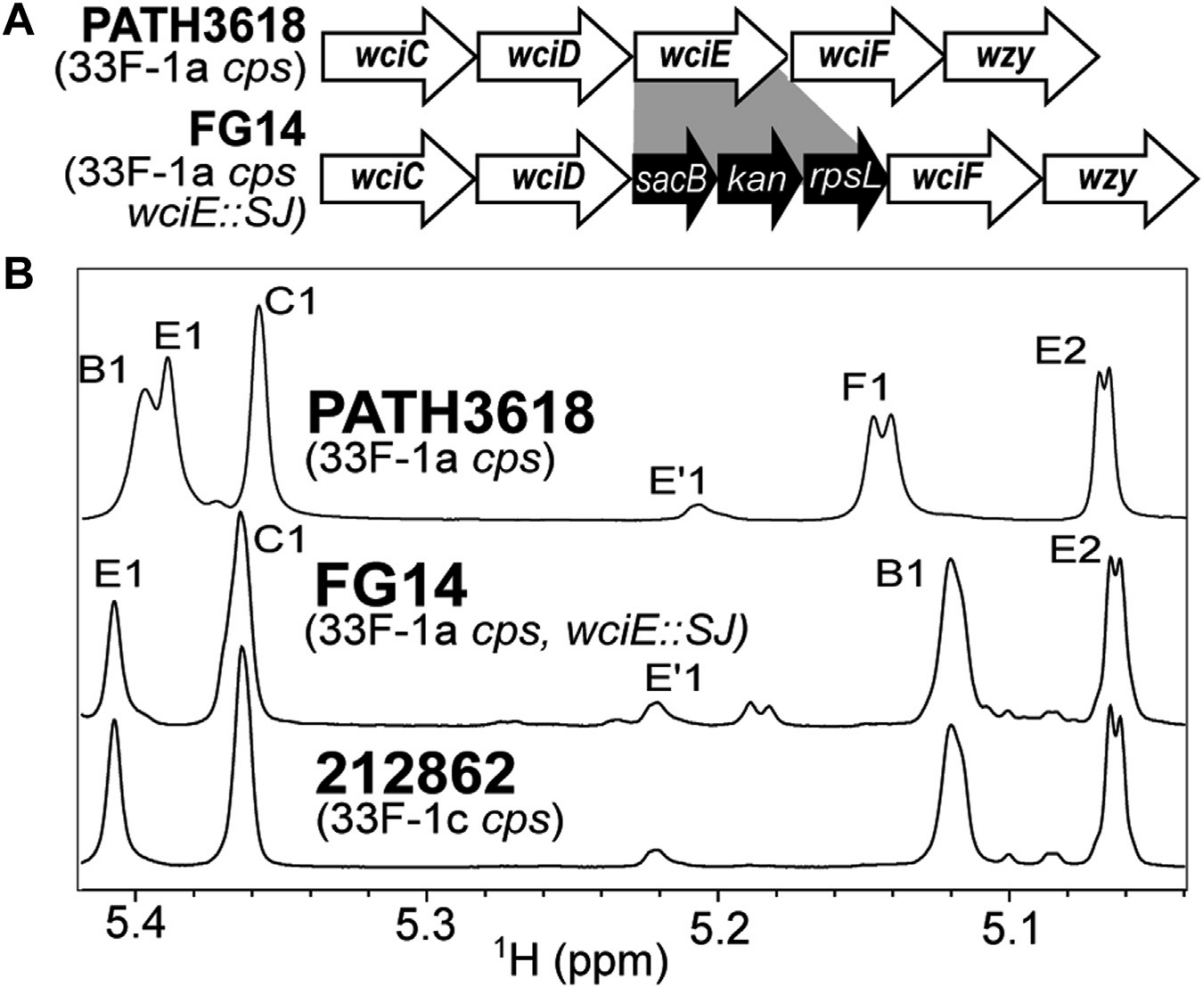
**Deletion of *wciE* results in loss of branching αGal*p* from 33F PS.***A*, recombinant strategy using Sweet Janus cassette to create the PATH3618 *wciE* knock-out mutant, FG14. *B*, ^1^H NMR spectra of native capsule PS purified from PATH3618, FG14, and 212862. Signals arising from each monosaccharide residue are assigned as labeled in Figure 2, *C* and *D*, as well as in Table 1. Residue Eꞌ represents the de-O-acetylated form of glycosyl residue E. NMR, nuclear magnetic resonance; PS, polysaccharide.

The link between the nonsense mutation in *wciE* of 33F-1c *cps* loci (Fig. 1*B*), and the changes observed in the PS synthesized by 212862 strongly suggests that WciE is a 1-2-galactose galactopyranosyl transferase. However, others previously postulated without molecular confirmation that WciE catalyzes the β-D-Gal*f*-(1→3)-β-D-Gal*p* linkage in 33F (28). To evaluate its role in capsule synthesis, we created a *wciE*-deficient recombinant mutant of PATH3618, FG14 (Fig. 3*A*). The ^1^H NMR spectrum of native capsule PS purified from FG14 no longer contained a signal corresponding to residue F and was identical to that of 212862 (Fig. 3*B*), confirming that *wciE* encodes a GT that mediates the addition of the branching α-D-Gal*p* and that its inactivation in a 33F strain results in expression of the 33E capsule type.

### Capsule types 33F and 33E are distinguishable using an anti-33F monoclonal antibody

Having elucidated the PS structure and genetic basis of 33E, we investigated whether this novel capsule type is antigenically distinguishable from other serogroup 33 capsule types. First, we conducted agglutination assays to confirm the reactivity of study strains to the serotyping pool and factor sera conventionally employed for the identification of serogroup 33 capsules (Table 2). Consistent with prior findings (24), STREP33F, PATH3618, 212862, FG14, and the serotype 33F reference strain SSISP33F/2 reacted only to Pool T, Pool E, and factor serum 33b, *i.e.*, demonstrated the “33F” antigenic profile. We next performed a well-established flow cytometric serotyping assay (FCSA) (29, 30) to test reactivity with mAb previously made for the detection of serotype 33F (31). While mAb Hyp33FG1 recognized all tested strains except SSISP33C/2 (used as a negative control), mAb Hyp33FM1 bound to 33F strains STREP33F, PATH3618, and SSISP33F/2 but not to 33E strains 212862 and FG14 (Table 2 and Fig. S1). Thus, 33F PS contains antigenic determinants that are absent in 33E PS, and 33E is an antigenically distinguishable capsule serotype.

**Table 2:** Strains phenotypically characterized in this study

Strain	*cps* genotype	Reference factor sera reactivity								FCSA^a^		NMR^b^
		Pool T	Pool E	33b	33e	33f	6a	20b	Profile^c^	33FM1^d^	33FG1	
STREP33F	33F	[+]	[+]	[+]	--	--	--	--	**33F**	[+]	[+]	33F
212862	33F-1c	[+]	[+]	[+]	--	--	--	--	**33F**	--	[+]	33E
PATH3618	33F-1a	[+]	[+]	[+]	--	--	--	--	**33F**	[+]	[+]	33F
FG14	33F-1a, *wciE::SJC*^e^	[+]	[+]	[+]	--	--	--	--	**33F**	--	[+]	33E
Reference strains												
SSISP33F/2	na^f^	[+]	[+]	[+]	--	--	--	--	**33F**	[+]	[+]	na
SSISP33A/2	na	[+]	[+]	[+]	--	--	--	[+]	33A	na	na	na
SSISP33B/2	na	[+]	[+]	--	--	[+]	--	--	33B	na	na	na
SSISP33C/2	na	[+]	[+]	--	[+]	[+]	--	--	33C	--	--	na
SSISP33D/2	na	[+]	[+]	--	--	[+]	[+]	--	33D	na	na	na

a FCSA, flow cytometric serotyping assay using anti-33F monoclonal antibodies.

b Capsule PS structure as determined by nuclear magnetic resonance (NMR) analysis in this study.

c profile according to conventional serotyping criteria.

d The 33FM1 cell line is unfortunately lost. However, our laboratory has archived the hybridoma supernatant for future experiments.

e Recombination deletion of *wciE* gene with Sweet Janus Casette (SJC).

f na, not applicable/not determined; [+], positive; --, negative.

### Immunization with 33F PS elicits a weaker functional antibody response to 33E

**Figure 4:**
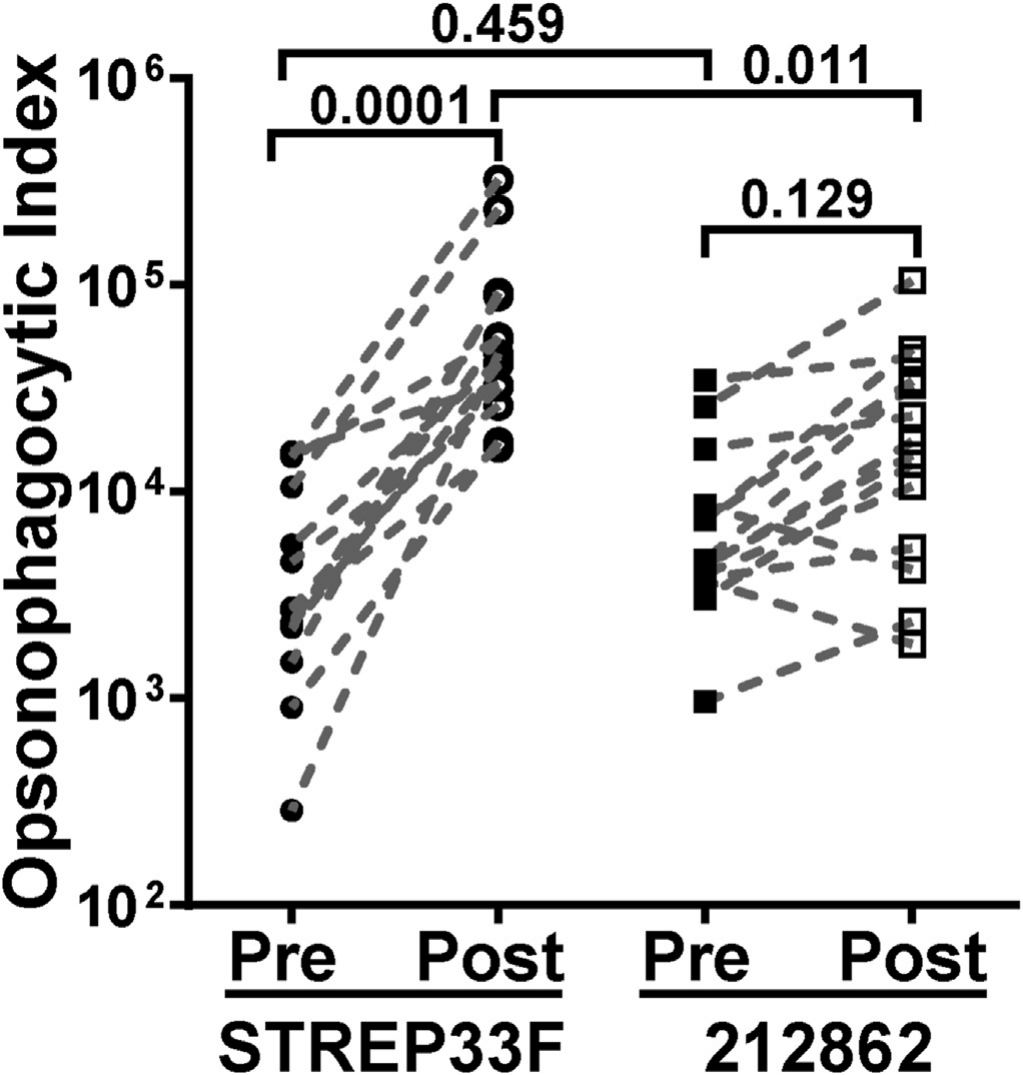
**Functional antibody response against 33F and 33E following immunization with 23-valent pneumococcal polysaccharide vaccine (PPSV23).** Opsonophagocytic index values against STREP33F (33F) and 212862 (33E) using matched preimmunization and postimmunization PPSV23 immunization sera samples from 14 adults. *p*-values are the results of a one-way analysis of variance (ANOVA) with Tukey’s multiple-comparison test.

To evaluate the potential role of 33E as a vaccine escape variant, we investigated whether immunization with the 23-valent pneumococcal PS vaccine containing 33F PS induces cross-reactive, functional antibodies to 33E in human adults (32). Paired preimmunization and postimmunization serum samples from six (Fig. S2) and 14 human individuals in two independent experiments were tested against STREP33F and 212862 targets in an *in vitro* opsonophagocytosis killing (OPK) assay (Fig. 4 and Table S2). This pilot study showed no significant difference in the absolute OPK indices for each strain. However, immunization resulted in a significant increase in OPK capacity against 33F (mean = 7.86 × 10^4^, *p* = 0.0001) but not to serotype 33E (mean = 2.57 × 10^4^, *p* = 0.129), supporting a more targeted functional response against 33F.

## Discussion

**Figure 5:**
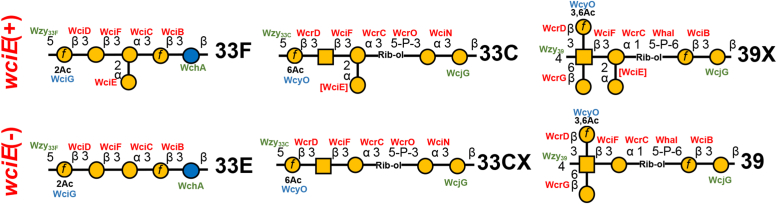
**Known and predicted capsule polysaccharide structures of *wciE* capsule-type variants.** Symbol Nomenclature for Glycan diagrams of the capsule PS repeat units of *wciE*-positive (*top row*) and *wciE*-negative (*bottom row*) capsule types. Structures predicted according to *cps* locus sequence analysis (see text) are denoted by an “X” in their names. *cps*-encoded glycosyl transferases (*red*), Wzy polymerases (*green*, *left*), initial transferases (*green*, *right*), and O-acetyltransferases (*blue*) are listed next to the structures they putatively synthesize, with brackets denoting enzymes whose presence is inferred according to analyses. The symbol key is found in Fig. 1*A*. *cps*, capsule synthesis; PS, polysaccharide.

Pneumococcal epidemiology and virulence vary widely according to capsule type (1, 33, 34), so being able to accurately detect different types is key to developing effective strategies against pneumococcal disease. We report the biochemical, genetic, and antigenic determinants of a newly discovered capsule type 33E. Though, 33E and 33F share very similar capsule PS structures, they can be distinguished serologically using the mAb Hyp33FM1. The 33FM1 epitope is likely dependent on the branching α-D-Gal*p*-(1→2)-α-D-Gal*p* structure on 33F PS and mediated by the *cps*-encoded GT WciE. Recombinant deletion of *wciE* in a 33F strain resulted in the expression of 33E, confirming the role of the naturally occurring nonsense *wciE* mutation in 33E *cps* loci. This finding prompted us to update the putative biosynthetic roles of enzymes encoded in the serogroup 33 *cps* loci (Fig. 5), though biochemical evidence is required to confirm these putative assignments.

The OPK is the primary measure of the immune response to pneumococcal vaccines in adults, and the OPK titers are the basis for vaccine licensure for older adults. Thus, OPK results play a crucial role in predicting vaccine effectiveness against a specific serotype. We detected differences between the response against 33F and 33E, elicited by immunization with 33F PS in adults, suggesting that the branching Gal*p* forms an immunodominant epitope in 33F. It is tempting to speculate that the antigenically distinct 33E capsule type may become more common as PCVs including 33F PS in their formulations become more widely implemented. Although further confirmation with a larger sample size is necessary in the future, our findings offer compelling evidence for vaccine developers to be aware of capsule type 33E and to distinguish it from 33F.

Though 212862 and the putative 33E isolate GPS_NZ_SPN11350 were obtained 2 years apart (2009 *versus* 2011) in different continents (Table S1), they are invasive disease isolates representing two independent genomic lineages and appear to have evolved through different *wciE* mutations in independent events during carriage with 33F precursors, akin to what has been observed with other capsule types (35). Additional 33E isolates will need to be evaluated to confirm whether 33E is capable of clonal propagation. 33E was detected among isolates containing the 33F-1 *cps* locus, but it is reasonable to assume that capsule type 33E can also emerge from strains harboring the canonical 33F *cps* locus like STREP33F. Indeed, an equally important finding in our study is that the 33F-1a isolate PATH3618 expresses 33F capsule, and presumptively all other isolates harboring the 33F-1a and 33F-1b *cps* loci (Table S1) does as well. Thus, while 33F-1 represents a phylogenetically distinct, global *cps* lineage that also encodes 33F capsule, “serotype 33F” isolates containing both 33F and 33F-1 *cps* loci should be equally screened for expression of 33E capsule, either by antibodies or *wciE* sequence analysis.

We initially hypothesized the existence of three 33F capsule variants and specifically evaluated for capsule PS harboring distinct O-acetylation patterns. Though *wcyO* is reported as a pseudogene in most of the 33F-1 *cps* loci (24, 26), a significant proportion (n = 18 of 30 GPS project isolates) harbor an intact *wcyO* (*i.e.*, 33F-1a). *cps*-encoded WcyO putatively mediates 6-O-acetylation of nonreducing end Gal*f* in multiple capsule types (*e.g.*, 33C and 39, Fig. 5) (28, 36); however, structural data obtained from the capsule PS purified from the 33F-1a isolate PATH3618 did not reveal any evidence of 6-O acetylation, indicating the presence of only a 33F O-acetylation pattern. We speculate that PATH3618 *wcyO*, despite sharing >99% nucleotide identity with *wcyO* from 33C, may harbor a missense mutation in an unidentified critical residue, rendering the gene nonfunctional. Alternatively, it is possible that the WciG-associated 2-O-acetylation of the nonreducing end Gal*f* present in most serogroup 33 capsule PS (Fig. 1*A*) may interfere with WcyO. This dominance of *wciG* masking the effect of *wcyO* may be related to the sequential expression of the acetyltransferase genes in the *cps* loci. Indeed, while various pneumococcal *cps* loci harbor up to three different O-acetyltransferase genes, no locus contains both *wciG* and *wcyO* (25). Despite the metabolic burden of modifying such a large superstructure, capsule O-acetylation is a conserved feature across various bacterial species and can greatly influence the epidemiological behavior of important human pathogens (33, 35). Thus, the mechanisms of capsule PS O-acetylation and its impact on bacterial physiology merits greater investigation.

A similar case can be made for branching monosaccharides on capsule PS. Akin to the 33F/33E capsule types, multiple cases of pneumococcal syntenic pairs differ according to the presence/absence of a branching monosaccharide and whether a GT *cps* gene is intact. The 7A *cps* locus contains a *wcwD* pseudogene which is intact in the homologous 7F *cps* locus (25). Accordingly, 7A capsule PS lacks a branching β-Gal*p* present in the otherwise identical 7F capsule PS structure (37, 38). The 19C PS structure is identical to 19B except that 19B lacks a branching β-Glc (39), likely as a result of 19B missing the GT gene *wchU*, which is present in the 19C *cps* locus (25). Inactivation of *whaF* in a 20B strain is associated with the loss of a branching α-Glc and putatively results in the expression of the 20A capsule type (10). Similarly, 23B and 23F *cps* loci harbor a putatively intact *wchW* gene (25); however, 23B PS lacks a branching α-rhamnose putatively mediated by *wchW* and is present in the otherwise identical 23F PS (40), so the genetic determinant is not readily apparent. Lastly, the 35A *cps* locus contains a *wcrK* pseudogene which is intact in the syntenic 35C *cps* locus (25) and likely determines the presence of a branching α-Glc present on 35C PS but absent from otherwise identical 35A PS (41).

Notably, there may be other unrecognized *wciE*-associated variants. Though 33C PS is reported to contain the *wciE*-dependent α-D-Gal*p*-(1→2)-α-D-Gal*p* (28), the reference 33C *cps* locus from strain 7098/41 completely lacks a *wciE* allele (25). It is possible that 7098/41 represents an unrecognized 33C variant (33CX), lacking the branching monosaccharide, and was mistyped as 33C (Fig. 5). Similarly, serotype 39 PS lacks an α-D-Gal*p*-(1→2)-α-D-Gal*p* branch (36), and the reference type 39 *cps* locus from strain 203/40 contains a *wciE* pseudogene (25), suggesting the existence of a *wciE*-positive variant (39X) that contains the branching monosaccharide (Fig. 5). Though these predicted capsule variants require confirmatory studies, it is worth assuming that branching monosaccharides are an underappreciated source of capsule structure variation. Moreover, studies have recognized that WciE belongs to a GT-32 family (PF05704) (Fig. S3) and is a retaining GT (42), that probably adds the branching α-Gal*p* residue on the cytoplasmic face. It has been demonstrated that side chain residue is essential for the proper assembly and processing of the capsule, and the inability to synthesize or process a complete RU is detrimental to the cell (43). Similar to O-acetylation (5, 29, 41, 44, 45), branching monosaccharides appear to be a capsule PS feature that can be readily lost (or gained) without disrupting overall capsule production. Coupled with the fact that branching monosaccharides can comprise the immunodominant epitopes of capsule PS (40), serotyping and bioinformatic surveillance tools should be continuously refined in anticipation that these features will be a major source of capsule variants emerging in response to ongoing immunization efforts.

Discovery and characterization of novel capsule types directly affect immunization efforts. Given that conventional methods mistype 33E as 33F, it is possible that the 33E is more prevalent than what is represented in this limited cohort of strains. Furthermore, pneumococci are under constant selective pressure to alter capsule expression in response to varying environmental forces or intrahost factors, and we can reasonably predict the presence of undiscovered capsule types among global populations. There are regular reports of immunized individuals having a disease caused by a vaccine serotype, *i.e*., cases of “vaccine failure” (46, 47). Coupled with the observation that functional antibody response to immunization can vary significantly to very similar capsule types, as shown here and in other studies (8, 13), it is possible that undiscovered capsule variants are responsible for a portion of observed vaccine failure cases. Therefore, one must remain skeptical of the ability of current typing methods to detect the emergence of relevant capsule variants, and surveillance tools should be adjusted to account for the mechanisms through which novel capsule types may arise.

## Experimental procedures

### Bacterial strains and cultivation

Pneumococcal strains GPS_US_PATH3618 (PATH3618) from Bangladesh (isolation year, 2006) and 2009212862 (212862) from the United States (isolation year, 2009) were obtained and characterized as a part of the GPS project (https://www.pneumogen.net/gps/), as previously described (24). Reference strains SSISP33A/2, SSISP33B/2, SSISP33C/2, SSISP33D/2, and SSISP33F/2 were obtained from Statens Serum Institut (SSI). STREP33F was previously described and housed in the Nahm Laboratory strain collection (27). Unless noted otherwise, strains were cultured on blood agar plates supplemented with 5% sheep blood (Remel Laboratories) or Todd-Hewitt broth with 5% yeast extract (THYb) and incubated at 37 °C in 5% CO_2_. Bacteria stocks were stored in THYb with 15% glycerol at −80 °C. The pneumococcal identity of all strains was confirmed using colony morphology, blood agar plate hemolytic activity, and optochin susceptibility (48, 49).

### Whole genome sequencing

Genomic DNA was extracted from STREP33F using a Monarch Genomic DNA purification kit (New England Biolabs). DNA library construction and sequencing were performed by SeqCenter. Raw reads were assembled into draft genomes using the *de novo* assembler Unicycler v0.4.7 [30]. Raw reads and assembled contigs are available on NCBI under BioProject PRJNA931299. Scaffolds.fasta files were used for downstream analysis.

### Comparative genetic analysis

Genetic sequences used in our analysis and their descriptions and accession numbers are listed in Table S1. Nucleotide and amino acid sequences were compared, translated, and analyzed by Geneious prime v2020. Multiple Alignment using Fast Fourier Transform was run with a scoring matrix of 200 PAM/K of 2 and a gap open penalty of 1.5.

### Construction of pneumococcal strain FG14

Mutant strain FG14 was constructed by recombinant deletion of *wciE in the cps locus* of PATH3618 using the Sweet Janus cassette strategy (50) (Fig. 3*A*). Briefly, upstream and downstream regions flanking *wciE* were PCR-amplified from PATH3618 genomic DNA using primers listed in Table S3. The flanking fragments and a Sweet Janus cassette were assembled into a single construct by overlap extension PCR (51). Purified amplicons were transformed into PATH3618, and transformants were selected on THY agar with 400 μg/ml kanamycin. Genomic recombination was confirmed by Sanger sequencing performed at the Heflin Center Genomics Core Lab at the University of Alabama at Birmingham.

### Capsule PS purification

Capsule PS was purified from strains PATH3618, 212862, STREP33F, and FG14, as described previously (6, 7). Ten milliliter of the culture of each strain was inoculated into 1 L of a chemically defined medium (52) supplemented with choline chloride (1 g/L), sodium bicarbonate (2.5 g/L), and cysteine HCl (0.73 g/L) and incubated at 37 °C for 16 h without shaking. Following centrifugation (15,344*g*, for 30 min at 4 °C) and removal of the supernatant, bacterial pellets were lysed by incubation in 0.9% aqueous NaCl containing sodium deoxycholate (0.05%) and mutanolysin (100 U/ml), for 72 h at 37 °C. Lysates were centrifuged, dialyzed against 4 L of 5 mM Tris (pH 7.3) with 3500-molecular-weight cutoff dialysis tubing, and applied to a DEAE Sepharose (GE Healthcare) anion exchange column. Serogroup 33 capsule PS are uncharged and do not bind to DEAE. Instead, capsule PS was recovered in flowthrough and wash fractions. Capsule PS-containing fractions were detected by anthrone assay (53). We pooled fractions containing high levels of capsule PS, but low levels of teichoic acid as determined by inhibition-ELISA testing binding of a phosphocholine-specific monoclonal antibody, HPCG2b (29, 54), to plates coated with pneumococcal teichoic acid (SSI), and other nonglycan contaminants were detected *via* absorbance at 260 and 280 nm. Pooled fractions were lyophilized and stored at −20 °C until analyzed.

### NMR spectroscopy

Approximately, 5 mg of capsule PS samples were dissolved in 0.6 ml of 99.99% D_2_O (Cambridge Isotope Laboratories). NMR data were collected at 35 °C on Bruker Avance III-HD (^1^H, 600 or 850 MHz) spectrometers equipped with cryogenic triple-resonance probes. ^1^H NMR spectrum was obtained by water suppression using a presaturation pulse sequence (zgpr). Complete assignment of ^1^H and ^13^C signals was achieved by two-dimensional nuclear Overhauser spectroscopy, correlation spectroscopy, total correlation spectroscopy, heteronuclear multiple quantum coherence, and heteronuclear multiple bond correlation spectra. NMR data were processed with NMRPIPE (55) and analyzed with NMRVIEW (56). HDO signal was used as a reference.

### Serological analysis

Slide agglutination and FCSAs were performed as previously described (6, 7, 41, 57). The following panel of polyclonal rabbit antisera was obtained for slide agglutination from the SSI: Pool T, Pool E, and factor sera 33b, 33e, 33f, 6a, and 20b. For FCSA, we used hybridoma supernatants containing Hyp33FG1 or Hyp33FM1 murine mAbs for the detection of the 33F capsule. Briefly, frozen bacterial stocks were thawed, washed, and incubated in FCSA buffer (phosphate-buffered saline, 3% fetal bovine serum, 0.1% NaN_3_) containing 1:5 dilutions of hybridoma supernatants for 30 min at 4 °C. After washing, bound immunoglobulin (Ig) was stained with 1:200 dilution of phycoerythrin-labeled anti-mouse Ig antibody (Southern Biotech) in FCSA buffer and detected by flow cytometry using BD Accuri C6 Plus (BD Biosciences) and FCS Express software.

### Opsonophagocytosis assay

A well-characterized UAB opsonophagocytosis assay (58, 59) (and described in detail at https://www.vaccine.uab.edu/uploads/mdocs/UAB-MOPA.pdf) was performed to investigate whether the 33F serotype elicits cross-opsonizing antibodies to serotype 33E. In two independent experiments, opsonophagocytosis assay was performed with six pairs and 14 pairs of pre- and post-PPSV23 vaccinated immune sera using serotypes 33F (STREP33F) and 33E (212862) as targets. Briefly, 30 μl of bacteria suspended in OBB (Hanks’ buffer supplemented with 0·1% gelatin and 5% fetal calf serum) was mixed with 10 μl of baby rabbit serum of specified concentration, and 40 μl of differentiated HL60 cells (10^7^ cells/ml) in OBB. The mixture was incubated with shaking (700 rpm) for 45 min at 37 °C with 5% CO_2_. Ten microliters from each well were spotted on THY agar plates, and the bacterial colonies were counted after overnight incubation.

## Data availability

Whole genome sequencing data (*i.e.*, raw reads and assembled contigs) of STREP33F are available on National Center for Biotechnology Information (NCBI) under BioProject PRJNA931299. All other data are contained within the manuscript and supporting information.

## Supporting information

This article contains supporting information (24, 26).

## Conflict of interest

U. A. B. has Intellectual Property rights on some reagents used in the study. F. A. G., J. S. S., J. J. C., and M. H. N. are U. A. B. employees. The authors declare that they have no conflicts of interest with the contents of this article.
