# Supplementary material for: Novel pneumococcal capsule type 33E results from the inactivation of glycosyltransferase WciE in vaccine type 33F
Source: J Biol Chem. 2023 Jul 24;299(9):105085. doi: 10.1016/j.jbc.2023.105085 (PMC10462825; doi:10.1016/j.jbc.2023.105085)
Supplement: Supporting Figures S1–S3 and Tables S1–S3 [file mmc1.docx]

**Supporting information for:**

**Novel pneumococcal capsule type 33E results from the inactivation of glycosyltransferase WciE in vaccine type 33F**

Feroze A. Ganaie^1^, Jamil S. Saad^2^, Stephanie W. Lo^3^, Lesley McGee^4^, [Andries J. van Tonder](https://journals.asm.org/doi/10.1128/mBio.00937-20#con4)^5^, Paulina Hawkins^4,6^, Juan J. Calix^1,7^, Stephen D. Bentley^3^, Moon H. Nahm^1,#^

^1^ Department of Medicine, Division of Pulmonary/Allergy/Critical care, University of Alabama at Birmingham, Birmingham, AL, USA

^2^ Department of Microbiology, University of Alabama at Birmingham, Birmingham, AL, USA

^3^ Parasites and Microbes, Wellcome Sanger Institute, Hinxton, Cambridge, UK

^4^ Respiratory Diseases Branch, Centers for Disease Control and Prevention, Atlanta, GA, USA

^5^ Department of Veterinary Medicine, University of Cambridge, Cambridge, United Kingdom

^6^ Hubert Department of Global Health, Rollins School of Public Health, Emory University, Atlanta, Georgia

^7^ Department of Medicine, Division of Infectious Diseases, University of Alabama at Birmingham, Birmingham, AL, USA

# **Corresponding Author:** Moon H. Nahm ([mnahm@uabmc.edu](mailto:mnahm@uabmc.edu))

**This file includes:**

- **Figure S1, S2, and S3**
- **Table S1, S2, and S3**


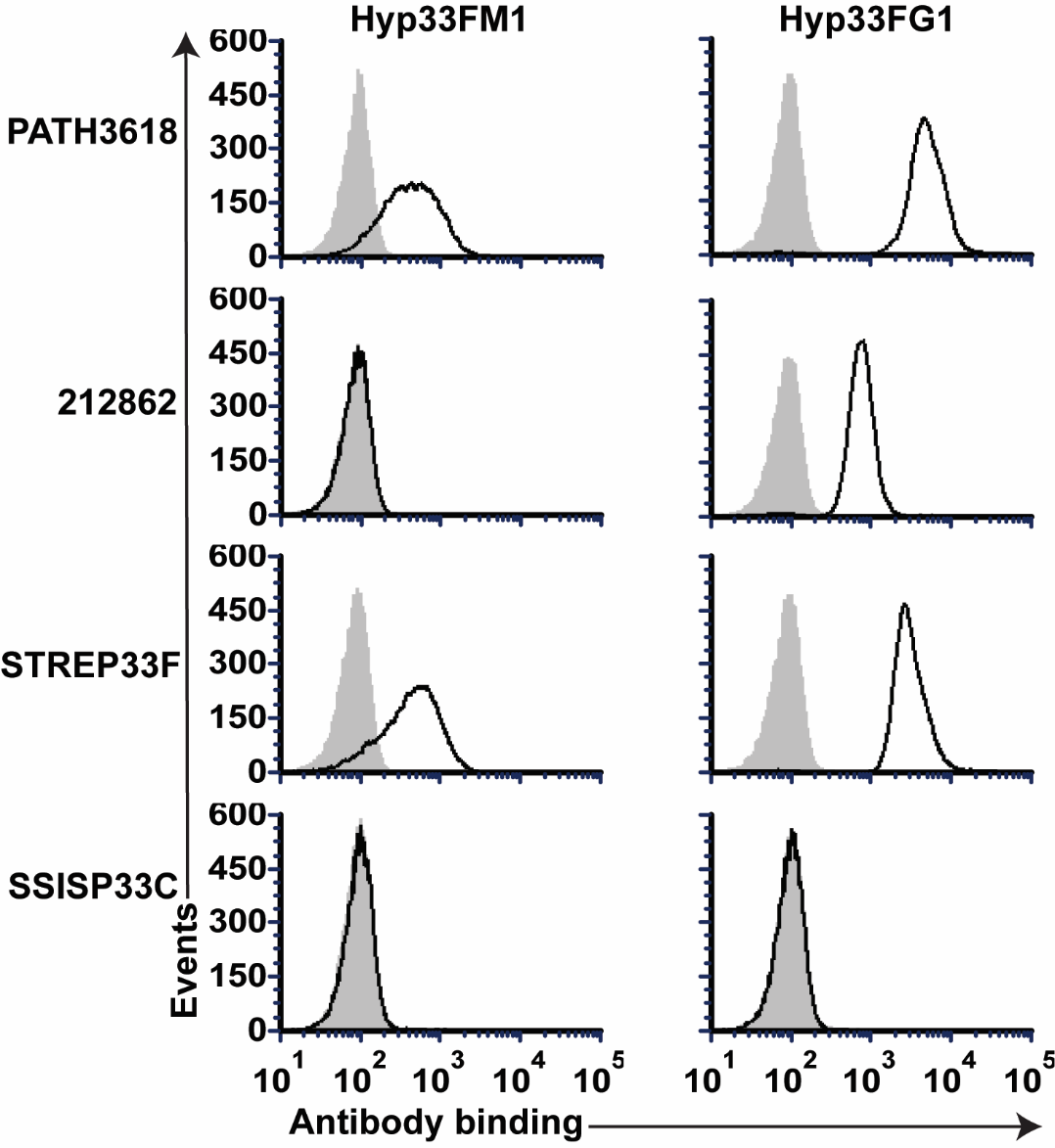


**Figure S1. Serological properties of representative serogroup 33 strains by flow cytometry analysis.** Histograms show the fluorescence of a bacterial strain (indicated to the left of each row of histograms) after staining with the in-house monoclonal antibodies (mAb’s) (indicated at the top of histograms). Solid black lines indicate the fluorescence obtained with primary and secondary antibodies; gray shaded areas indicate control binding (secondary antibody alone). The x-axes show the log fluorescence intensity (antibody binding), and the y-axes, the number of events (cell counts). Each strain was tested 3 times, and representative results are shown.


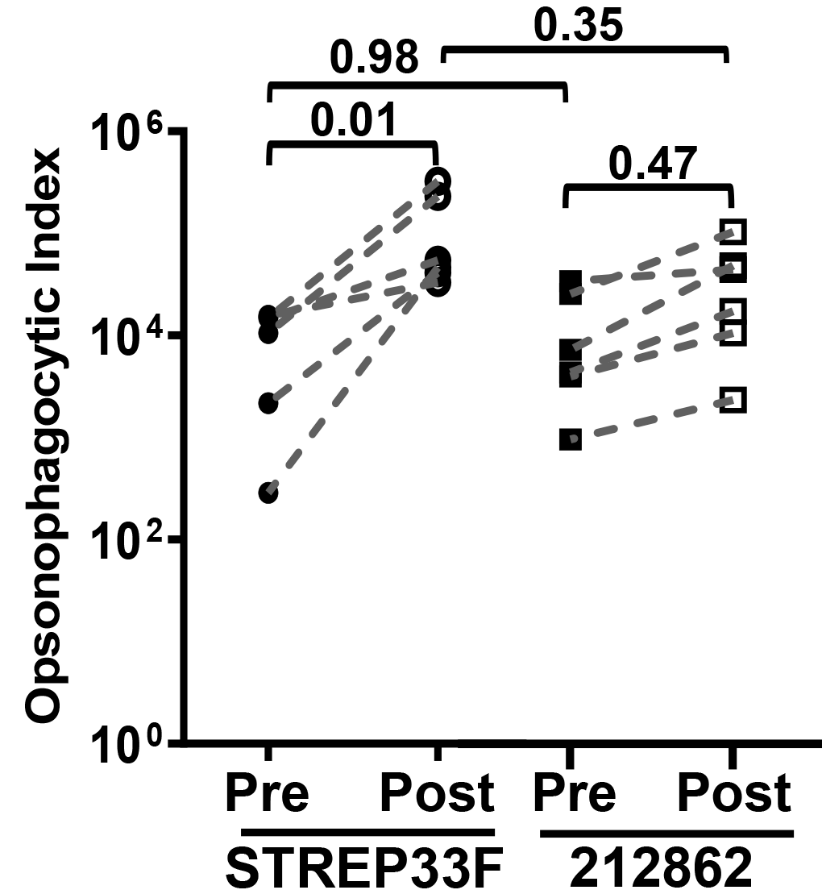


**Figure S2. Functional antibody response against 33F and 33E following immunization with 23-valent pneumococcal polysaccharide vaccine (PPSV23).** Opsonophagocytic index values against STREP33F (33F) and 212862 (33E) using matched pre- and post-immunization PPSV23 immunization sera samples from six adults. *p*-values are the results of a one-way analysis of variance (ANOVA) with Tukey’s multiple-comparison test.


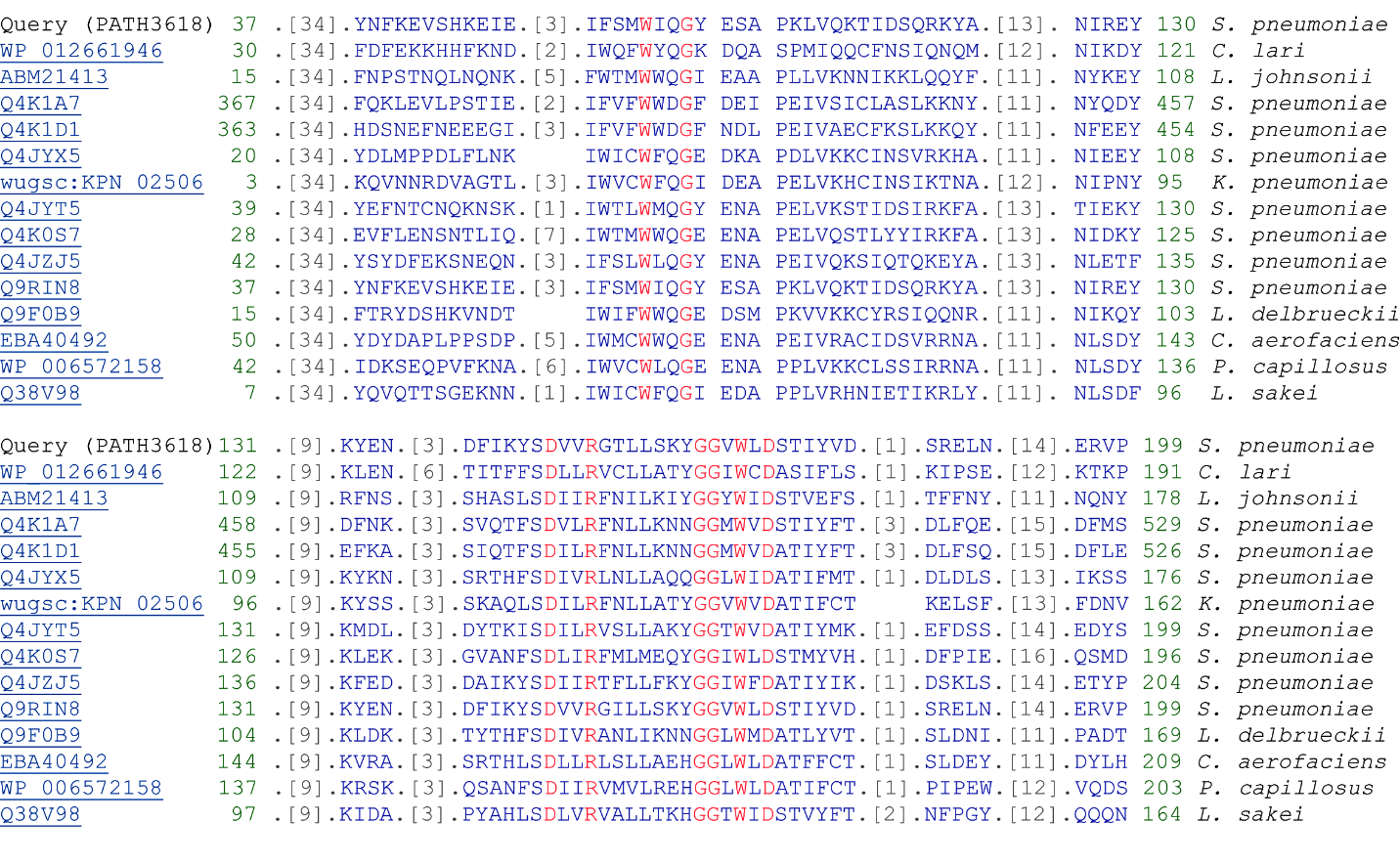
**Figure S3. Alignment of putative pneumococcal WciE and WciE-like glycosyltransferases (GTs) belonging to GT-32 family.** The alignment shows the putative conserved amino acids (red color) critical for the function of WciE and WciE-like GTs. For clarity purposes, amino acids not shown in the alignment are represented by numbers in square brackets in gray color. The amino acid position in the polypeptide chain is indicated by numbers in green color. The protein ID of each putative GT is indicated on the left side of the alignment and the species name is indicated on the right side of the alignment. Query sequence refers to the WciE amino acid sequence of PATH361.

**Table S1. 33F-1 genomes evaluated in this study**

| **Strain** | **Isolate information** | | | |  | | |  | | **Genomic Information** | | | | |  |  |
| --- | --- | --- | --- | --- | --- | --- | --- | --- | --- | --- | --- | --- | --- | --- | --- | --- |
|  | **Type** | **Year** | **Country** | **Serotype^1^** | | **MLST** | ***cps* type** | | ***wciE*** | | ***wcyO*** | **Accession number** | | **Reference** | | |
| PMP1348 | NP carriage | No data | Fiji | 33F | | 13802 | 33F-1b | | Intact | | Truncated | MH256127 | | (26) | | |
| PMP1349 | NP carriage | No data | Fiji | 33F | | 13802 | 33F-1b | | Intact | | Truncated | MH256128 | | (26) | | |
| PMP1351 | NP carriage | No data | Fiji | 33F | | 13802 | 33F-1b | | Intact | | Truncated | MH256129 | | (26) | | |
| PMP1352 | NP carriage | No data | Fiji | 33F | | 13802 | 33F-1b | | Intact | | Truncated | MH256130 | | (26) | | |
| PMP1353 | NP carriage | No data | Fiji | 33F | | 13802 | 33F-1b | | Intact | | Truncated | MH256131 | | (26) | | |
| PMP1379 | NP carriage | No data | Fiji | 33F | | 13802 | 33F-1b | | Intact | | Truncated | MH256132 | | (26) | | |
| PMP1380 | NP carriage | No data | Fiji | 33F | | 13802 | 33F-1b | | Intact | | Truncated | MH256133 | | (26) | | |
| PMP1883 | NP carriage | No data | Fiji | 33F | | 13802 | 33F-1b | | Intact | | Truncated | MH256134 | | (26) | | |
| PMP1386 | NP carriage | No data | Mongolia | 33F | | 673 | 33F-1b | | Intact | | Truncated | MH256135 | | (26) | | |
| PMP1387 | NP carriage | No data | Mongolia | 33F | | 673 | 33F-1b | | Intact | | Truncated | MH256136 | | (26) | | |
| **GPS isolates** | | | | | | | | | | | | |  | | |  |
| GPS_ZA_1761 | Invasive | 2010 | South Africa | 33F | | 673 | 33F-1a | | Intact | | Intact | ERR730764 | | (24) | | |
| GPS_US_PATH3618 | Invasive | 2006 | Bangladesh | 33F | | 6044 | 33F-1a | | Intact | | Intact | ERR750820 | | (24) | | |
| GPS_US_PATH3655 | Invasive | 2009 | Bangladesh | 33F | | 6044 | 33F-1a | | Intact | | Intact | ERR750853 | | (24) | | |
| GPS_MW_C7253_R1 | Invasive | 2003 | Malawi | No data | | 10608 | 33F-1a | | Intact | | Intact | ERR774628 | | (24) | | |
| GPS_MW_19149 | NP carriage | No data | Malawi | No data | | 12260 | 33F-1a | | Intact | | Intact | ERR869889 | | (24) | | |
| GPS_NP_1281 | NP carriage | 2009 | Nepal | 33F | | 7573 | 33F-1a | | Intact | | Intact | ERR980286 | | (24) | | |
| GPS_HK_310 | NP carriage | 2010 | China | 33F | | 673 | 33F-1a | | Intact | | Intact | ERR1453638 | | (24) | | |
| GPS_IN_P40 | Invasive | 2014 | India | 33B | | 12815 | 33F-1a | | Intact | | Intact | ERR1756404 | | (24) | | |
| GPS_IL_23574 | Invasive | 2009 | Israel | 33F | | 673 | 33F-1a | | Intact | | Intact | ERR2089756 | | (24) | | |
| GPS_IL_26736 | Invasive | 2013 | Israel | 33F | | 673 | 33F-1a | | Intact | | Intact | ERR2090654 | | (24) | | |
| GPS_IL_26764 | Invasive | 2013 | Israel | 33F | | 673 | 33F-1a | | Intact | | Intact | ERR2090656 | | (24) | | |
| GPS_IL_27161 | Invasive | 2013 | Israel | 33F | | 673 | 33F-1a | | Intact | | Intact | ERR2090847 | | (24) | | |
| GPS_IL_27600 | Invasive | 2014 | Israel | 33F | | 673 | 33F-1a | | Intact | | Intact | ERR2090830 | | (24) | | |
| GPS_IL_24759 | Invasive | 2010 | Israel | 33F | | 673 | 33F-1a | | Intact | | Intact | ERR2091113 | | (24) | | |
| GPS_IL_25775 | Invasive | 2011 | Israel | 33F | | 673 | 33F-1a | | Intact | | Intact | ERR2091161 | | (24) | | |
| GPS_IL_26186 | Invasive | 2012 | Israel | 33F | | 673 | 33F-1a | | Intact | | Intact | ERR2091186 | | (24) | | |
| GPS_IL_26655 | Invasive | 2013 | Israel | 33F | | 673 | 33F-1a | | Intact | | Intact | ERR2091300 | | (24) | | |
| GPS_NP_2047 | NP carriage | 2012 | Nepal | 33F | | 7573 | 33F-1a | | Intact | | Intact | ERR979949 | | (24) | | |
| GPS_IN_P65 | Invasive | 2015 | India | 33B | | 7573 | 33F-1b | | Intact | | Truncated | ERR1756414 | | (24) | | |
| GPS_GM_3037 | Invasive | 2015 | Cameroon | No data | | 10608~ | 33F-1b | | Intact | | Truncated | ERR2089167 | | (24) | | |
| GPS_GM_3076 | Invasive | 2015 | Cameroon | No data | | 10608~ | 33F-1b | | Intact | | Truncated | ERR2089214 | | (24) | | |
| GPS_NP_6511 | NP carriage | 2014 | Nepal | 33F | | 7538 | 33F-1b | | Intact | | Truncated | ERR980330 | | (24) | | |
| GPS_MW_BHA2WF_R1 | Invasive | 2013 | Malawi | No data | | 12260 | 33F-1b | | Intact | | Truncated | MK606435 | | (24) | | |
| GPS_MW_BQN1FT | NP carriage | 2014 | Malawi | No data | | 10608 | 33F-1b | | Intact | | Truncated | ERR862485 | | (24) | | |
| GPS_MW_BQN1D8 | NP carriage | 2014 | Malawi | No data | | 10608 | 33F-1b | | Intact | | Truncated | ERR862480 | | (24) | | |
| GPS_NP_1008 | NP carriage | 2009 | Nepal | 17F | | 7538 | 33F-1b | | Intact | | Truncated | ERR980224 | | (24) | | |
| GPS_NP_1013 | NP carriage | 2009 | Nepal | 6 | | 7538 | 33F-1b | | Intact | | Truncated | ERR980226 | | (24) | | |
| GPS_NP_1041 | NP carriage | 2009 | Nepal | 33F | | 7538 | 33F-1b | | Intact | | Truncated | ERR980232 | | (24) | | |
| 2009212862 | Invasive | 2009 | USA | 33F | | 60 | 33F-1c | | Truncated | | Truncated | ERR433945 | | (24) | | |
| GPS_NZ_SPN11350 | Invasive | 2011 | New Zealand | 33F | | 673 | 33F-1c | | Truncated | | Intact | ERR1788088 | | (24) | | |

^1^ according to conventional serotyping methods reported in original studie

**Table S2.** **Opsonophagocytic killing titers against 33F and 33E capsule types using paired pre- and post-PPSV23 immunization sera**

| Pre vaccine | | | Post vaccine | | |
| --- | --- | --- | --- | --- | --- |
| Sample ID | **OPK Titer^ǂ^** | | **Sample ID** | **OPK Titer^ǂ^** | |
|  | **STREP33F** | **212862** |  | **STREP33F** | **212862** |
| MD175 | 1499 | 3813 | MD177 | 92012 | 5352 |
| MD176 | 2204 | 4683 | MD198 | 88262 | 32746 |
| MD319 | 905 | 7318 | MD354 | 17422 | 34373 |
| MD209 | 2668 | 16143 | MD230 | 16360 | 23340 |
| MD006 | 5551 | 8640 | MD016 | 42394 | 4194 |
| MD181 | 2343 | 3045 | MD195 | 55810 | 13704 |
| MD251 | 4589 | 3699 | MD270 | 26043 | 1837 |
| MD063 | 2749 | 4471 | MD088 | 32230 | 15102 |
| P32A_01 | 287 | 969 | P32C_01 | 46434 | 2360 |
| P016A_02 | 2171 | 4014 | P016B_02 | 41249 | 10644 |
| P37A_03 | 10551 | 7320 | P37C_03 | 231508 | 49005 |
| P014A_04 | 14817 | 25783 | P014B_04 | 323441 | 104957 |
| P31A_05 | 15634 | 34538 | P31C_05 | 33097 | 44658 |
| P30A_06 | 14624 | 4363 | P30C_06 | 54542 | 17496 |

STREP33F (33F capsule type) and 212862 (33E capsule type); OPK, opsonophagocytic killing.

^ǂ^OPK titers against STREP33F (33F) and 212862 (33E) were calculated using matched pre- and post-immunization PPSV23 immunization sera samples from 14 adults.

**Table S3. Primers used in this study**

| Primer name | Sequence | Target region |
| --- | --- | --- |
| 51300 | ttaacagtagttgtaccatcatataatgcagaaaattatttacaagag | 5´ flanking region |
| 31064 | ATTATCCGGTACCTCTCctcgtttattacttttcctattttcctcatattatttccttatcccc |  |
| 51301 | gaaaagtaataaacgagGAGAGGTACCGGATAATGCTGAAAACTCC | Sweet Janus |
| 31065 | tcatcaatctatcaaaGAGACTCGAGCCTTTCCTTATGCTTTTGGA |  |
| 51302 | AAAGGCTCGAGTCTCtttgatagattgatgaagggtgaactgtagcataaagagag | 3´ flanking region |
| 31066 | catcttatcttttattttatagtagtaatttggggaaagcagaaaca |  |

**References** (used in the main text)

24. van Tonder, A. J., Gladstone, R. A., Lo, S. W., Nahm, M. H., du Plessis, M., Cornick, J., Kwambana-Adams, B., Madhi, S. A., Hawkins, P. A., Benisty, R., Dagan, R., Everett, D., Antonio, M., Klugman, K. P., von Gottberg, A., Breiman, R. F., McGee, L., Bentley, S. D., and The Global Pneumococcal Sequencing, C. (2019) Putative novel *cps* loci in a large global collection of pneumococci. *Microb Genom* **5**, e00027

26. Manna, S., Dunne, E. M., Ortika, B. D., Pell, C. L., Kama, M., Russell, F. M., Mungun, T., Mulholland, E. K., Hinds, J., and Satzke, C. (2018) Discovery of a *Streptococcus pneumoniae* serotype 33F capsular polysaccharide locus that lacks *wcjE* and contains a *wcyO* pseudogene. *PLoS One* **13**, e0206622
